# Synthesis and Performance of TiO_2_/Fly Ash Cenospheres as a Catalytic Film in a Novel Type of Periodic Air-Sparged Photocatalytic Reactor

**DOI:** 10.3390/ma13071691

**Published:** 2020-04-04

**Authors:** Witold Żukowski, Przemysław Migas, Dariusz Bradło, Piotr Dulian

**Affiliations:** Faculty of Chemical Engineering and Technology, Cracow University of Technology, ul. Warszawska 24, 31-155 Cracow, Poland; przemyslaw.migas@pk.edu.pl (P.M.); dariusz.bradlo@pk.edu.pl (D.B.); piotr.dulian@pk.edu.pl (P.D.)

**Keywords:** catalytic film, titanium dioxide, cenospheres, photocatalytic reaction

## Abstract

The results of a photocatalytic process performed in a new type of inclined, three-phase fluidised bed reactor with a periodic photocatalyst film are presented. These phases were fly ash cenospheres coated with TiO_2_, an aqueous solution of methylene blue and an air stream passing from the bottom of the photoreactor. The cenospheres have a density lower than water and could thus form a catalytic film on a top irradiated window. The formed surface film is stable but is easy to break and be reproduced in a cyclic air-sparged process. Mixing was performed in either a cyclic or a continuous manner. From an operational point of view, the best variant of mixing was a 10 s air-sparge/10 s break with a 50% duty cycle, because it provided the same discolouration efficiency and reduced energy demand by 50% in comparison with the continuous mixing. Due to film formation, the proposed catalytic reactor enables a substantial reduction in the energy required for mixing while maintaining the desired degree of discolouration.

## 1. Introduction

The application of the photocatalytic processes is one of the methods of water purification. It can be used when biological methods fail, for example, in the treatment of wastewater containing high concentrations of phenol [1]. There are also combined methods in which photocatalysis supports other wastewater treatment processes, such as a heterogeneous Fenton-like reaction [2]. Titanium dioxide is one of the most commonly used compounds in photocatalytic reactions; this is primarily as a result of its relatively low cost and chemical stability [3]. Titanium dioxide can also be coupled with other compounds or ions in order to increase its photocatalytic activity [2,4,5,6,7,8,9,10]. Nevertheless, the key factor in effective photocatalytic water purification, apart from the type of catalyst (decisive for the kinetics and the light wavelength allowing excitation), is photoreactor construction and the way in which the photocatalyst is distributed in the reaction zone. This is especially important from the point of view of optimal photocatalyst irradiation as well as the mass exchange between the surface of the photocatalyst and the reaction zone. A frequently used solution in photocatalytic reactors is the impregnation of the photocatalyst on its walls [11,12,13]; however, this type of reactor suffers from a low surface area of effective photocatalyst and low rates of mass transfer. The dispersion of the photocatalyst in the reaction zone is ensured in the other type of photoreactor, where the catalyst is either used in the form of a TiO_2_ suspension [14,15] or it is deposited on a special support. The latter case is advantageous due to costs associated with the separation of post-process water and a catalyst (i.e., centrifugation and sedimentation) being avoided. Moreover, different solutions for the organisation of photocatalytic processes are presented in the literature, inter alia, optical fibre covered with photocatalyst, microreactors, and reactors where the photocatalyst is immobilised on the irradiated rotating surface, a water-bell photoreactor, etc. [16,17,18]. When the photocatalyst is deposited on the support, three variants can be distinguished in terms of support density: it can be lower, higher, or similar to that of water and then the catalyst floats on the water surface, sinks or is suspended within the water, respectively. From the point of view of performance, the best option is the first because the use of such a catalyst obviously ensures effective catalyst distribution in the reaction zone and provides optimal light utilisation. Usage of a photocatalyst with a density below 1 g/cm^3^ is very common, for example, when perlite, cork, or polymers are used as photocatalytic support [19]. In this work, fly ash cenospheres were used, which are characterised by their hollow and spherical structure, low apparent density and high mechanical resistance [20].

One of the problems connected with photoreactors is the requirement for an appropriate mixing system in order to ensure the proper dispersion of a photocatalyst, which may be enforced by using two- or three-phase fluidised bed systems which provide much more efficient mass exchange compared to reactors with a fixed film [1,21]. The other issue is related to optimal reactor surface utilisation and this could be achieved through the usage of inclined reactors, as presented in the literature, i.a. [17,18,22]. An example of such a solution is an inclined PTC reactor (parabolic trough collector), in which the photocatalytic material in the form of slurry is dispersed inside a transparent tube that is located in the focal area of a concave mirror. One of the existing variants of this solution is the PROPHIS (parabolic trough-facility for organic photochemical syntheses in sunlight) — parabolic trough reactor that operates under sunlight [23]. Castilla-Caballero et al. [24] modelled a similar type of photoreactor as a series of plug flow reactors (PFRs) and continuous stirring tanks (CSTRs), in order to obtain the theoretical knowledge for scaling-up purposes. The reactor developed by Bousselmi et al. which cleans water from textile industry dyes is yet another example of this solution [11,12]. In this variant, TiO_2_ is immobilised on the outer surface of the reactor.

The flat inclined fluidised bed reactor that offers both cyclic and continuous aeration is used in this paper, and studies on the effect of periodic mixing on the overall time of dye degradation and energy balance were performed. The conducted tests are the starting point for the development of an effective method of photocatalytic wastewater treatment, where photocatalyst is deposited on a support with a density below 1 g/cm^3^, especially on cenospheres. The use of a suspended catalyst involves ensuring the best possible catalyst distribution in the reaction space and ensures the best possible irradiation. In the literature, neither a comparable solution for running the photocatalysis process nor similar studies on the effect of periodic mixing on the overall degradation time and the energy balance of the process were found.

## 2. Materials and Methods

### 2.1. Materials

Fly ash cenospheres (Połaniec Coal Power Plant, Poland) were used as the catalytic support. Raw cenospheres were subjected to purification from small fly ash as well as porous and broken particles by heating in water (90 °C, 1 h). The floating fraction was then dried (105 °C, 12 h) and screened to obtain a grain fraction of 200–250 µm. Titanium isopropoxide (TTIP) with a purity of 97% (Sigma-Aldrich, Saint Louis, MO, USA) was the titanium source. In the experiments, 5 ppm of methylene blue (MB) solution was made from pure substance (Hadron Scientific, Kielce, Poland).

### 2.2. Catalyst Preparation

**Figure 1:**
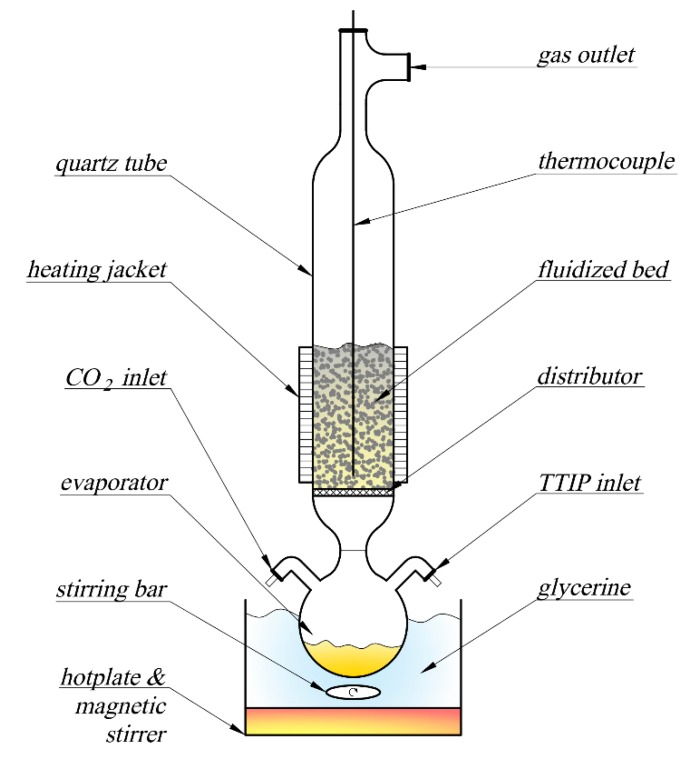
Fluidised-bed chemical vapour deposition (FB-CVD) experimental setup.

The FB-CVD (fluidised-bed chemical vapour deposition) method was used for deposition of the active phase onto the cenosphere support (Figure 1). The fluidised bed was formed from 30 g of fly ash cenospheres, and carbon dioxide was the fluidising gas, which provided inert conditions in the reactor and prevented decomposition of TTIP below the distributor. The flow rate of CO_2_ was maintained at a level of 0.04 m/s using a Dwyer GFC-1111 mass flow meter. TTIP was introduced to an evaporator and its vaporisation rate was adjusted using a glycerine bath at a temperature of 100 °C. The temperature of the bed was set at 400 °C by heating a resistance jacket connected with a thermocouple and a Lumel RE82 programmable temperature controller. After complete vaporisation of the TTIP, the CO_2_ was replaced by air and the temperature was raised to 500 °C for 30 min. Burnout of carbonaceous residuals followed by TiO_2_ recrystallisation occurred in these conditions. The final mass gain of the bed was 3.8 wt.% (approx. 75% yield) and the product had an apparent density of around 0.8 g/cm^3^, thus it could easily float on the surface of the water.

### 2.3. Characterisation of Materials

The crystal structure of the prepared titanium dioxide layers was determined using X-ray diffraction (XRD) using a PW1830 X-ray diffractometer (Philips, Amsterdam, Netherlands) with a CuKα radiation source (λ = 1.54056 Å) for 2θ = 10–70° with a step size of 0.05. Scanning electron microscope (SEM) images of the microstructure and morphology of the obtained material were taken with a TM-3000 SEM microscope (Hitachi, Chiyoda, Tokio, Japan). Optical photographs were obtained by means of a TPL Trino (Rhede, Germany) stereoscopic microscope equipped with a DLT-Cam PRO 5 MP camera (Mińsk Mazowiecki, Poland).

### 2.4. Photocatalytic Experiments

**Figure 2:**
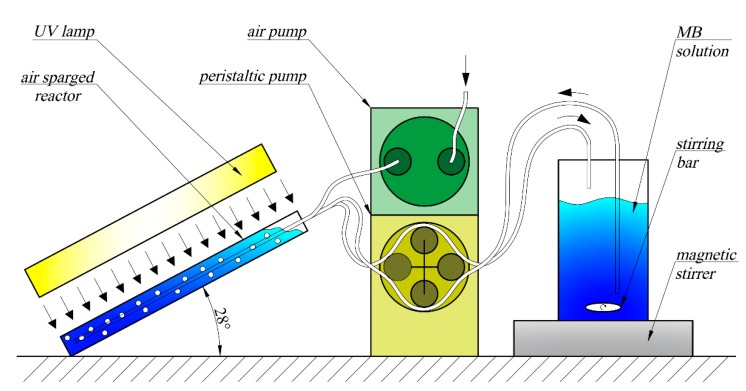
Laboratory system scheme.

**Figure 3:**
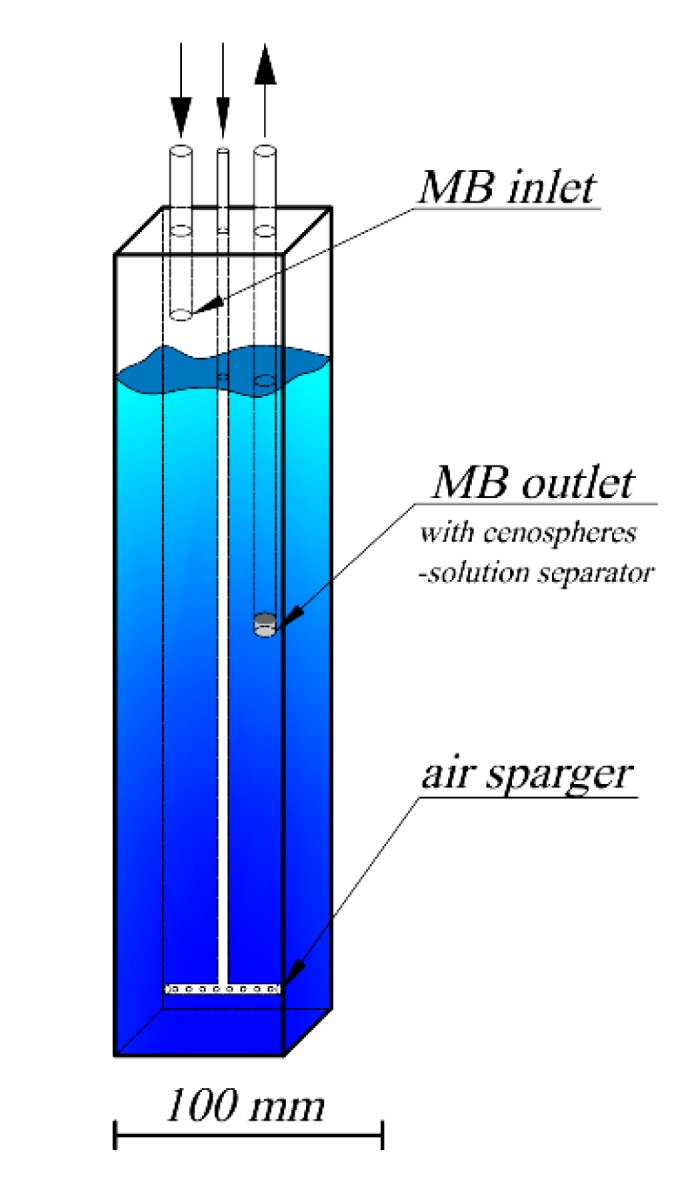
Geometry of air-sparged reactor and scheme of media inlets and outlets.

The experiments were conducted in the inclined photoreactor (Figure 2 and Figure 3), in which side walls were made from window glass and the front wall was made from type JGS2 quartz glass (Conductance Company, Ostrów Wielkopolski, Poland). Inclination of the reactor is advantageous in relation to both vertical and horizontal arrangements. In the case of a vertical reactor, the area of irradiation is lower, while a horizontal reactor generates difficulties with controlling the direction of the gas flow. The optimal solution was obtained when inclination was applied, and it provided appropriate distribution of the active catalytic phase as well as mixing the whole reaction volume. The volume of the reaction zone (irradiated volume) was around 160 cm^3^, whereas the total volume of the methylene blue (MB) solution in the circuit was 750 cm^3^. The irradiated area (front wall surface) was around 133 cm^2^. A catalyst was introduced into the reactor at the amount of 0.5, 1 or 2 g, depending on the experiment. Furthermore, the photolysis process without catalyst addition was examined. The MB solution was introduced to the reactor using a peristaltic pump with a volumetric rate of 30 cm^3^/min via an inlet tube located above the reaction zone and it was drained through an outlet tube terminating with a separator (a 60 µm sieve that separated cenospheres from the solution). The average residence time of the solution in the reactor was around 5 min. Depending on the experiment, the reactor was mixed either cyclically or continuously with an air flow of around 100 cm^3^/s. Three mixing modes where used: continuous mixing (100% duty cycle—labelled 10/0), 10 s air-sparge/10 s break (50% duty cycle—labelled 10/10), and 10 s air-sparge/20 s break (33% duty cycle—labelled 10/20).

In the initial trials with visible-light irradiation of the examined photocatalyst, no activity was observed. Before starting the UV lamp, the system was mixed without using dark surroundings but in a darkened environment in the fume cupboard (using the 10 s air-sparge /20 s break mode) for about 90 min to establish sorption equilibrium. After this time, the MB concentration reached equilibrium, which was taken as a reference value in further calculations. The 18 W UV-C lamp was then turned on (located approx. 5 cm above the reactor) and the appropriate mixing mode was set. Discolouration of the model sewage during the experiment (every 40 min, 2 cm^3^ MB solution was withdrawn from the reactor) was determined using the spectrophotometric method at a wavelength of 665 nm.

### 2.5. Process of Catalytic Film Formation and Breaking

**Figure 4:**
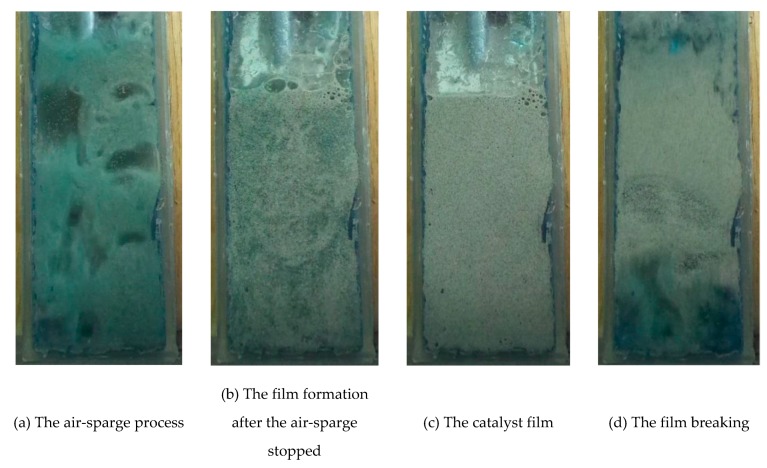
The cyclic process of the catalytic film formation and breaking.

Figure 4 shows the process of mixing the MB solution with the cenosphere catalyst forced by cyclic aeration, as well as the processes of formation and breaking of the catalytic film. The film formation was, as mentioned above, possible due to the inclination of the reactor (at an angle of 28°). When the air-sparge is provided, the cenospheres are dispersed in the reaction zone. After stopping the air-sparge, the cenospheres locate near the inclined wall made of quartz.

### 2.6. Kinetic Calculation

The discolouration process was assumed to have the pseudo first order kinetic:

(1) $$\frac{C_{MB}}{C_{MB0}}=e^{-k\times \tau }\;,$$

where τ is the time of the reaction (min); $C_{MB}$ is the methylene blue concentration in time *τ* (ppm); $C_{MB0}$ is an initial methylene blue concentration (ppm); *k* is the reaction rate constant, calculated from a regression (1/min).

On the basis of the rate constant, both the half time and the relative energy consumption for the mixing process (to achieve 50% MB degradation) were calculated:

(2) $$\tau_{0.5}=\frac{ln2}{k\;}\;\left[min\right],$$

(3) $$E=\frac{D\times \tau_{0.5i}}{\tau_{0.5\;r}}\;,$$

where *E* is the coefficient of energy consumption; $\tau_{0.5\;r}$ is the half time (min) for the reference experiment (2 g catalyst and full-time stirring); $\;\tau_{0.5i}$ is the half time for an i-th experiment (min); *D* is the duty cycle of the single aeration signal and is expressed by the following equation:

(4) $$D=\frac{t_{S}}{t_{S}+t_{R}}\;,$$

where $t_{R}$ is the duration of the mixing break period (0 s, 10 s or 20 s) and $t_{S}$ is the duration of the catalyst mixing period in the cycle (10 s).

Efficiency of catalyst utilisation (U*)* was calculated from the following equation:

(5) $$U=\frac{\tau_{0.5\left(1g\right)}\times 1g\;}{\tau_{0.5\left(m\right)}\times m}\times 100\%\;,$$

where $\tau_{0.5\left(1g\right)\;}$ is the half time for experiments where 1 g of catalyst was used and subscript *m* is the mass of the catalyst.

## 3. Results

**Figure 5:**
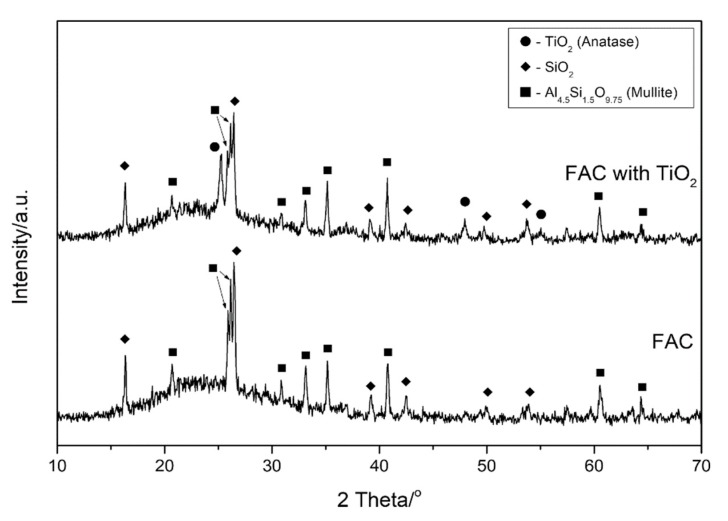
Diffractograms of fly ash cenospheres (FAC) and FAC with TiO_2_ coating.

**Figure 6:**
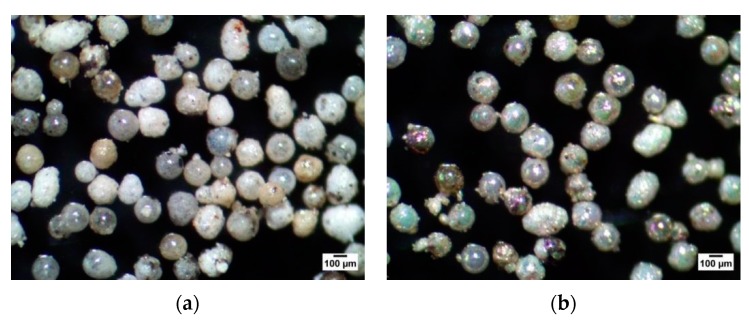
Microscopic photographs of tested materials (40 ×): (**a**) FAC, (**b**) FAC with TiO_2._

**Figure 7:**
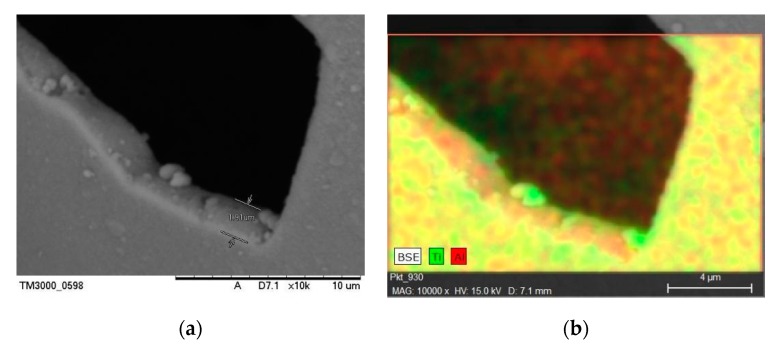
Scanning electron microscope (SEM) microphotographs of FAC with TiO_2_: (**a**) SEM image, (**b**) energy-dispersive X-ray spectroscopy (EDS) mapping.

Diffractograms of fly ash cenospheres (FAC) are presented in Figure 5. The clearly visible increased background level in the range of 15–35° 2 Theta suggests a significant contribution of the glassy amorphous phase. The dominant crystalline phases present in the cenospheres are mullite and quartz. In the sample of FAC with TiO_2_, an additional anatase phase was detected, thus proving the formation of a titanium oxide coating on the surface of the cenospheres. Furthermore, colourful reflections typical of nanometric transparent oxide layers were observed under the microscope (Figure 6b). It is worth noting that no agglomerates of this material could be observed for the FB-CVD method in contrast to the most commonly used sol-gel technique. The SEM image with the energy-dispersive X-ray spectroscopy (EDS) of a prepared cenosphere particle (Figure 7) confirmed the successful application of titanium dioxide on the cenosphere surface and its homogenous distribution.

**Figure 8:**
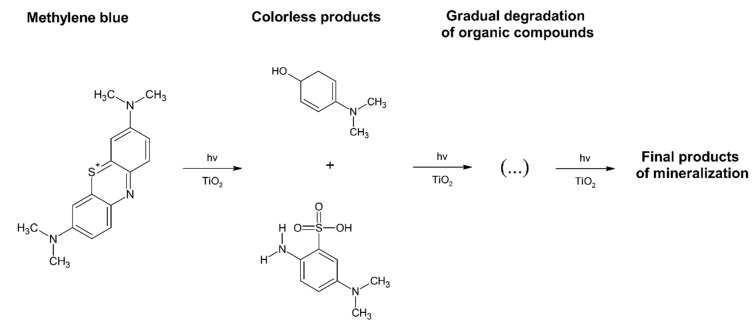
Simplified scheme of the methylene blue (MB) degradation by photocatalytic process on TiO_2_ coatings under ultraviolet (UV) irradiation [27,28].

Based on the literature, it is stated that the photocatalytic mineralisation of methylene blue is a process in which the first stage is dye decolourisation. Methylene blue is a complex compound which consists of three condensed aromatic rings (Figure 8, left) and substituents that affect polarity and the ability to adsorb electromagnetic radiation in the visible light range. It is clear that the degradation of such a compound will take place in several stages [25,26]. Products with smaller molecular weights and more reactivity will gradually be formed due to the gradual disappearance of condensed structures, followed by the disappearance of individual aromatic rings.

On the other hand, it is known from studies on the properties of TiO_2_ (anastase) that free radicals such as OH^.^ are formed in aqueous solution after absorption of photons on the photocatalytic surface [27]. These active radicals have a high oxidizing potential and oxidize other compounds in a non-selective way, thus if they are created, it leads to the complete decomposition of all organic compounds in aqueous solution resulting from the first step of methylene blue degradation.

The resulting products generated at the first step of degradation (decolorization) undergo further decomposition reactions with these active radicals formed on the photocatalyst. Thus, the methylene blue mineralisation process can be shown schematically in Figure 8 [27,28].

According to the scheme shown above, the proof of the catalytic activity of the produced material is the course of the first reaction, which results in discoloration of the dye solution. This allows the selection of the spectrophotometric method for analysing the MB degradation process and assessment of the photocatalytic activity of the tested material.

**Figure 9:**
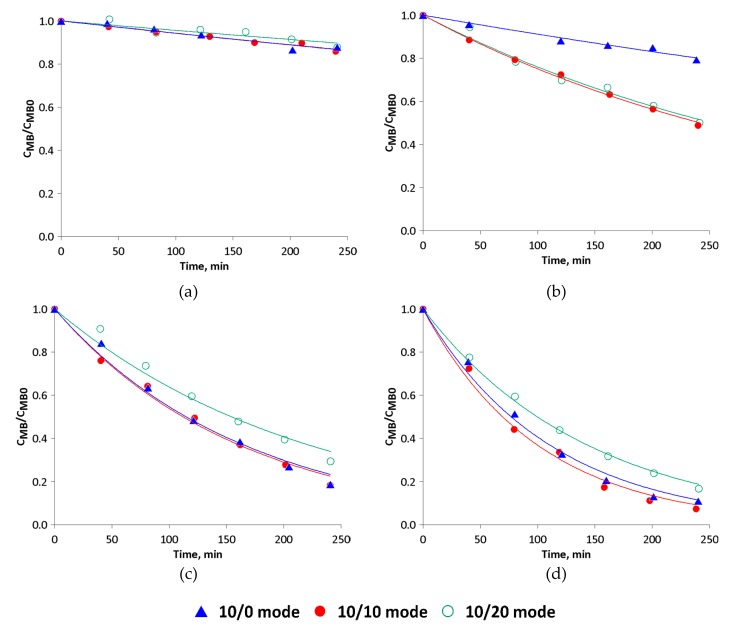
MB concentration changes with experiment time for different catalyst masses: (**a**) 0 g (photolysis), (**b**) 0.5 g, (**c**) 1 g, (**d**) 2 g.

Figure 9 shows the relative concentration of MB in the solution as a function of time. A value of 1 is related to the relative concentration after the sorption process. The highest degree of discolouration was achieved in the test in which 2 g of catalyst was used, when for each test after 240 min, the solution concentration decreased to below 20% of its initial value. The photolysis process (0 g of catalyst) led to linear discolouration over time, and no differences in the applied mixing mode were observed. The final solution concentrations (after ~240 min) were around 85% of the initial value.

The presented results show that when the low mass of the catalyst was applied (0.5 g refers to 0.67 g/L) in the continuous mixing mode (10/0), only a slight improvement in discolouration was observed in comparison to photolysis (around a 20% MB concentration reduction), which is connected with a high dispersion level of catalyst particles and the effect of light irradiation hindrance by the solution volume. Surprisingly, when mixing was periodically stopped, the efficiency of the process dramatically increased. This fact unambiguously proved that the formation of a catalytic film is advantageous. The time of the mixing breaks (10 s or 20 s) does not influence the process with this catalyst’s mass. On the contrary, the tests with 10/10 mixing mode led to similar results in comparison to 10/0 mode tests for higher catalyst masses, while tests with the 10/20 mixing mode are characterised by a lower rate of solution discolouration in each case.

The phenomenon described above can be explained by the antagonistic action of two factors: light irradiation availability and diffusion. Firstly, when the catalyst forms a film onto the front wall of the reactor, the barrier (in the form of a solution) between the light irradiation and the catalytic layer almost disappears. Thus, the efficiency of the catalytic reaction rate is at the highest possible level due to light irradiation availability. Simultaneously, stopping mixing leads to a lower diffusion rate of the compounds from the solution to the catalyst surface and in the opposite direction, and as a result, it reduces the purification rate. When analysing these two factors, it could be concluded that in continuous mode (10/0), when the diffusion rate is the highest, light irradiation availability is insufficient even for 1 g of catalyst. Conversely, in the 10/20 mode, the increase in reaction rate caused by the reduction of the solution barrier does not compensate for the decrease in reaction rate due to a decrease in turbulence. This suggests in turn that the long duration of film stabilisation (during periods of time without gas flow) does not lead to an increased rate of photocatalytic reaction, which is much faster than diffusion. The optimal conditions in terms of discolouration rate are ensured only when short time breaks in mixing are provided, when increased irradiation can degrade compounds adsorbed on the catalyst surface and the diffusion rate is not significantly disrupted by breaks in mixing.

It is important to note that the double mass of the catalyst does not proportionally increase discolouration efficiency, even in the continuous mixing mode (10/0). This implies that catalyst particles hinder the light irradiation to other particles both in the bulk solution and on the film, where only the monolayer surface can be irradiated. Therefore, there is also some optimal amount of catalyst, around 1 g, which provides maximal usage of the catalytic surface. On the other hand, when the catalyst is rather inexpensive, as is the case with the proposed catalyst, the higher increase in its amount would lead to further improvements in the catalytic process efficiency up to the point where particles would completely block light irradiation to the bulk liquid.

The discolouration efficiency is characterised by the photocatalytic reaction rate or time required to purify a certain portion of waste, e.g., 50% of initial concentration ($\tau_{0.5}$). However, the overall efficiency of the process should also take into consideration the operational cost in terms of energy utilisation. In the proposed set-up, turning off mixing saves energy in the form of pump operation.

The half-life $\tau_{0.5}$ (Table 1) increases with decreases to the photocatalyst mass. For 2 g of the catalyst and 10/10 mixing mode, it is the lowest and is 69 min, while in the 10/20 mode, it rises to 100 min, which is an increase of approximately 44%. This is obviously unfavourable, because to maintain similar discolouration in a certain period of time, it would mean an increase in the photocatalytic surface (reactor area). Reducing the catalyst mass causes a considerable time increase in the range of 112–154 min and 252–748 min in the case of 1 g and 0.5 g, respectively. 

When the half-time is calculated per 1 g of catalyst, some interesting information about the degree of catalyst utilisation can be made. When the further assumption is made that the process with 1 g of the catalyst is the reference, it turns out that in the process using 2 g of the photocatalyst, around 20% of the catalytic particles are not involved in the reaction, irrespective of the mixing modes. These numerical values can easily illustrate the aforementioned observations based on kinetic curves. What is more, in the process using 0.5 g of catalyst with continuous mixing, only around 30% of the catalyst is active; however, when mixing occurs and film is present, the usage of photocatalyst is near 100% in 10/10 mode. An alternative or complementary explanation of the observed results can be provided by multistep diffusion of the irradiation phenomenon. UV light undergoes scattering (diffraction, dispersion) before reaching the catalyst surface. In the case of the continuous mixing mode, when no film is formed, irradiation is scattered by thick layers of solution, thus its action is substantially weakened. On the other hand, in the variant when a catalytic film is formed, the light scattering occurs on the particles of the photocatalyst. When its film is too thick (as is the case in the experiment with 2 g of catalyst), only part of the photocatalyst is active.

It is worth noting that in the 10/20 mode with 0.5 g of catalyst, the time needed to reduce MB concentration to 50% (calculated per 1 g) is the lowest; this suggests that there is incomplete utilisation of the photocatalyst in this mode for higher catalyst masses. An explanation of the obtained observations could be the existence of a depletion layer around the surface of the photocatalyst. It is possible that some motionless fluid phases with a high concentration of MB degradation products are formed on the catalytic surface. This depletion layer could then be one of the causes of the decrease in process efficiency when long mixing breaks were applied (10/20 mode). Unfortunately, the measurement of compound concentration in a selected area of the reaction volume is complicated. In this paper, only the degree of total discolouration was examined, thus it was not possible to confirm the formation of the depletion layer.

**Figure 10:**
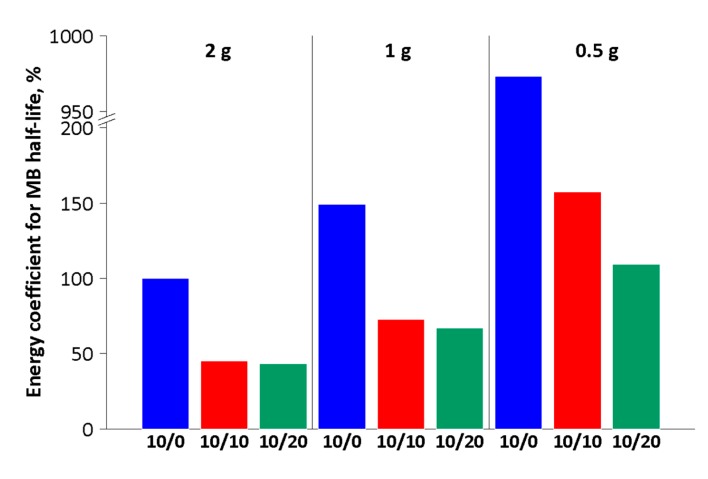
Relative energy demand of the mixing process to achieve a 50% dye reduction, depending on the mixing mode (Note: Ordinate axis was broken at 200%).

In order to evaluate the relative energy demand of a given process, the experiment with 2 g of catalyst and continuous mixing (10/0) was taken as a reference (100%). Lowering the mass to 1 g led to an increase of around 50% in energy demand (Figure 10), but a further decrease of the catalyst amount caused a dramatic growth of energy consumption (to approx. 975%), which is connected with the aforementioned poor dispersion of the catalyst. The usage of periodic aeration causes at least a two-fold decrease in the energy cost of the mixing process, irrespective of the catalyst mass used. Surprisingly, the energy demand does not differ significantly in the 10/10 and 10/20 variants for experiments with 1 g or 2 g of the catalyst. Therefore, from a procedural point of view, it is most preferable to use the 10/10 mixing mode. In this case, similar rates of the discolouration process are achieved as for a system with continuous mixing, but a two-fold reduction of energy consumption for a mixing process could also be obtained.

## 4. Conclusions

The use of an inclined three-phase fluidised photocatalytic reactor with a periodic mixing of the cenospheric bed enables reductions to the energy cost of the mixing process while maintaining the desired degree of discolouration. Through breaking and formation of the cyclical film, the reactor combines the features of a reactor with a suspended and an immobilised photocatalyst. The catalyst film formation period is characterised by a significant reduction of the solution layer located between the source of light irradiation and the catalytic surface. As a result, this leads to an improvement in photocatalyst exposure. Simultaneously, interruption of mixing leads to a decrease in the rate of mass transfer between the solution and the catalyst surface, thus slowing the purification process. The obtained results proved that the generation of a catalytic film was beneficial and the time of its formation (~4 s) is probably the optimal time for mixing breaks.

From the operational point of view, the best variant of mixing was 10 s air-sparge/10 s break (10/10) with a 50% duty cycle because it provided the same discolouration efficiency and reduced the energy consumption by half in comparison to continuous mixing. The best amount of catalyst was 2 g because it required around 60% less time or energy to purify the MB solution in comparison to 1 g in the 10/10 mixing mode. However, this value is not optimal because of incomplete (80%) catalyst utilisation. On the other hand, utilisation of an inexpensive catalyst based on waste material as a support enables usage of higher catalyst amounts with a reasonable economical balance.

## Figures and Tables

**Table 1:** Summary of the photocatalytic experiments using a periodic air-sparged reactor.

Mass of the Catalyst (g)	Mixing Mode	k (1/min)	R^2^	$\tau_{0.5}\;(\min)$	$\tau_{0.5}\cdot \mathrm{Mass},\;$(min × g)	U (%)
2	10/0	0.00902	0.996	77	154	75
	10/10	0.00999	0.996	69	139	81
	10/20	0.00696	0.997	100	199	77
1	10/0	0.00604	0.987	115	115	100
	10/10	0.00619	0.992	112	112	100
	10/20	0.00450	0.979	154	154	100
0.5	10/0	0.00093	0.977	748	374	31
	10/10	0.00287	0.997	242	121	93
	10/20	0.00275	0.980	252	126	122
photolysis	10/0	0.00055*	0.947	1251	—	—
	10/10	0.00056*	0.976	1239	—	—
	10/20	0.00043*	0.852	1608	—	—

* For 0-order kinetic.
